# Dose–response modelling uncovers compound‐specific inhibitory effects of host defence volatiles on mountain pine beetle fungal symbionts

**DOI:** 10.1002/ps.71060

**Published:** 2026-06-25

**Authors:** Duo Zhang, Yanzhuo Liu, Xinyi Zhao, Guncha Ishangulyyeva, Nadir Erbilgin

**Affiliations:** ^1^ Department of Renewable Resources University of Alberta Edmonton AB Canada; ^2^ College of Veterinary Medicine Jilin University Changchun China

**Keywords:** *Dendroctonus ponderosae*, plant–insect–fungi interaction, beetle–fungus symbiotes, dose–response modelling, secondary metabolites, monoterpene

## Abstract

**BACKGROUND:**

The mountain pine beetle relies on symbiotic fungi to overcome host tree defences, yet these fungi must tolerate the toxic volatile compounds produced by their hosts. However, how individual volatiles affect fungal symbionts and whether these effects constrain fungal performance when exposed to such toxic compounds remain poorly understood. Quantifying the responses of mountain pine beetle fungal symbionts to host defence volatiles can help explain growth constraints within beetle–fungus symbioses in host trees.

**RESULTS:**

We evaluated the toxicity of eight pine defence volatiles, seven monoterpenes and one phenylpropene, 4‐allylanisole, on the three primary fungal symbionts, *Grosmannia clavigera*, *Leptographium longiclavatum* and *Ophiostoma montium*, of the mountain pine beetle *in vitro*. Fungal cultures were exposed to a range of concentrations of each volatile, and individual growth responses were quantified to determine dose–response relationships. All volatiles reduced fungal growth, except for (−)‐α‐pinene against *L. longiclavatum*, although sensitivity varied substantially among fungal species and volatiles. *Grosmannia clavigera* was overall the most strongly inhibited. We found that toxicity differed between enantiomers. Among the volatiles tested, 4‐allylanisole and terpinolene were highly toxic to fungi, whereas (−)‐α‐pinene was the least inhibitory overall. Even with comparable concentrations at intermediate inhibitory potency, some volatiles (e.g. 3‐carene) required much higher concentrations to achieve near‐complete growth inhibition.

**CONCLUSION:**

These findings show that pine defence volatiles exert differential, compound‐specific impacts on the fungal symbionts of mountain pine beetles, suggesting that chemical diversity in tree defences may enhance their defences by simultaneously targeting multiple components of the beetle–fungus symbioses. © 2026 The Author(s). *Pest Management Science* published by John Wiley & Sons Ltd on behalf of Society of Chemical Industry.

## Graphical abstract

Host defence volatiles inhibit mountain pine beetle fungal symbionts in a compound‐ and enantiomer‐specific manner, with distinct dose–response profiles that reveal the multitarget chemical defences of beetle–fungi symbiotes.

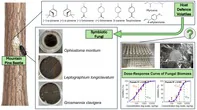

## 1 INTRODUCTION

The mountain pine beetle (MPB, *Dendroctonus ponderosae*, Coleoptera: Curculionidae, Scolytinae) has devastated millions of hectares of pine (*Pinus*) forests in western North America.^1^ A key to its success is a multipartite mutualism with fungi that it carries to new host trees.^2^, ^3^, ^4^, ^5^ In particular, MPB is associated with three main ophiostomatoid fungi: *Grosmannia clavigera*, *Leptographium longiclavatum* and *Ophiostoma montium*.^2^, ^3^ These symbiotic fungi benefit the beetle by helping it overcome host defences and providing nutritional support to developing larvae. However, the fungi differ in their virulence and ecological roles.^6^, ^7^, ^8^, ^9^ *Grosmannia clavigera* is often considered the most pathogenic partner, rapidly colonizing fresh phloem.^10^, ^11^ *Ophiostoma montium*, by contrast, is less virulent and tends to proliferate later after colonization of tissues by more aggressive symbionts in already defence‐weakened trees.^12^, ^13^ *Leptographium longiclavatum* shows intermediate characteristics between *G. clavigera* and *O. montium*, which are especially suitable in the cold environment, and can co‐occur with *G. clavigera* in northern MPB populations.^2^, ^14^, ^15^ These symbiotic fungi can compete with or coexist in the beetle's galleries, suggesting niche differentiation based on temperature and host chemistry.^12^, ^16^, ^17^

Pine trees employ potent chemical defences that challenge both the beetle and its fungi. Upon attack, conifers mobilize oleoresin, a sticky, toxic blend of secondary metabolites dominated by monoterpenes, diterpene acids and sesquiterpenes.^18^, ^19^ Because monoterpenes constitute a major and highly dynamic fraction of oleoresin and are rapidly released and oxidized at the wound site, they often represent the first and strongest chemical barrier encountered by attacking beetles and their fungal associates.^20^, ^21^, ^22^ The composition of these monoterpenes varies widely among pine species. For example, the oleoresin of lodgepole pine (*Pinus contorta*, the historical host of MPB) contains high proportions of monoterpene β‐phellandrene and terpinolene, whereas jack pine (*Pinus banksiana*, a novel host in the expanded range) is richer in monoterpene α‐pinene and 3‐carene.^23^, ^24^, ^25^ Such interspecific differences in monoterpene composition can influence a tree's susceptibility to MPB.^26^, ^27^, ^28^ In addition to monoterpenes, pines produce other allelochemicals such as the phenylpropanoid 4‐allylanisole, which is known to repel or disrupt bark beetles’ aggregation behaviour.^29^

The presence of symbiotic fungi fundamentally alters the host–beetle–fungal interaction. On the one hand, fungal infection can overwhelm the host by girdling stems and exhausting tree defences, thereby enhancing successful beetle establishment on host trees.^7^, ^30^ On the other hand, the fungi themselves are exposed to and must tolerate the host's toxic monoterpenes and other metabolites. The genome of *G. clavigera* encodes several cytochrome P450 monooxygenases and an efficient ABC transporter that confer resistance to monoterpenes.^10^, ^31^, ^32^ These symbiotic fungi also can metabolize monoterpenes, and *G. clavigera* and *O. montium* have even been found to utilize certain host defence volatiles as carbon and energy sources for growth.^32^, ^33^, ^34^ These suggest a coevolutionary arms race in which the tree produces toxins, and the beetle and their fungal symbionts, evolve countermeasures to thrive in chemically defended host trees.^9^, ^35^ The beetle–fungus symbionts may therefore circumvent some host defences because MPB's fungi can alter or sequester host defence volatiles, thereby reducing toxicity to the beetle.^9^ Host monoterpenes also can shape beetle–host interactions by acting as semiochemical precursors or cues. For example, (−)‐α‐pinene is toxic at high doses yet can be converted by MPB into the aggregation pheromone, (−)‐*trans*‐verbenol.^21^, ^36^ Likewise, 4‐allylanisole can inhibit the symbiotic fungi but has been reported to function as a beetle repellent or anti‐aggregation cue in some pines.^29^

Although the importance of host phytochemicals in bark beetle ecology is well‐recognized, studies have largely focused on beetles or tree defences, with relatively little emphasis on fungal symbionts.^35^ The toxicity of individual monoterpenes to adult MPBs has been quantified: for example, limonene is one of the most lethal monoterpenes to the beetle, whereas α‐pinene and terpinolene are markedly less toxic.^21^, ^22^ The effects of monoterpene concentration and mixtures on MPB performance also have been examined, showing that higher total monoterpene concentrations generally reduce beetle survival, although beetle traits can mitigate these effects.^22^, ^35^ By contrast, quantitative data on how these same host defence volatiles affect MPB fungal symbionts have been scarce. Some monoterpenes are known anecdotally or qualitatively to inhibit MPB fungal symbionts, but systematic comparisons across multiple fungi and host defence volatiles have been lacking.^20^, ^37^, ^38^, ^39^

Recent research is beginning to address this gap and suggests that different host defence volatiles may have distinct targets in beetle–fungus symbiosis. In lodgepole pine, natural variation in whole monoterpene profiles differentially affected MPB performance and the growth of its fungal symbiont.^35^ Likewise, Zaman *et al*.^40^ showed that no single monoterpene is maximally toxic to both MPB and its fungi. These findings raise the following questions: How do host defence volatiles vary in toxicity across different species of MPB's fungal symbionts? Do host defence volatile toxicity patterns of fungal symbionts align with (or contrast against) the toxicity patterns reported for adult MPB? As MPB expands into novel hosts with different resin chemistries (e.g. jack pine in Canada's boreal forest), understanding the susceptibility or tolerance of each symbiotic fungus to these new chemical environments is crucial and timely.^24^, ^26^

We systematically tested the toxicity of eight major pine host defence volatiles, including seven monoterpenes [(+)‐α‐pinene, (−)‐α‐pinene, (+)‐limonene, (−)‐limonene, 3‐carene, terpinolene, myrcene] and one phenylpropanoid (4‐allylanisole), on the three fungal symbionts of MPB: *G. clavigera*, *L. longiclavatum* and *O. montium*, *in vitro*. We exposed fungal cultures to a wide range of concentrations for each compound and measured growth inhibition to derive dose–response curves. The objectives of this research were to: (1) determine the extent to which each host defence volatile inhibits fungal growth (quantified by reductions in fungal biomass), (2) compare species‐specific tolerance and sensitivity by estimating toxicity parameters [the concentrations causing 50% (IC₅₀) and 90% growth inhibition (IC₉₀) and slope of inhibition response] for each fungus–volatile combination, and (3) evaluate the potential ecological implications of these tolerance differences in the context of MPB host colonization. By linking fungal performance to host chemical profiles, we aim to explain how host defences may differentially impact members of the beetle's symbiotic community, and how these interactions could shape the outcomes of beetle attacks in both historical and novel pine hosts.

## 2 MATERIALS AND METHODS

### 2.1 Fungal isolates and master plate preparation

Three MPB symbiotic fungal species were used in this study. *Grosmannia clavigera* isolate Tria544, originally isolated from Oregon (USA), was obtained from the University of British Columbia (Vancouver, Canada). *Leptographium longiclavatum* isolate JM8, originally isolated from Colorado (USA), was obtained from the USDA Forest Service Forest Products Laboratory (Madison, USA). *Ophiostoma montium* isolate USUME025, originally isolated in Alberta (Canada), was obtained from the Northern Forestry Centre, Canadian Forest Service (Edmonton, Canada). We established the master cultures by transferring mycelial plugs (7 mm diameter) from the actively growing edge of colonies onto potato dextrose agar (PDA). We prepared the PDA by dissolving 39 g PDA powder (Becton, Dickinson and Company, Sparks, MD, USA) in 1 L distilled water, followed by autoclaving at 121 °C for 20 min, and pouring 30 mL into each 100 mm × 15 mm polystyrene Petri dish. We then incubated cultures in darkness at 25 °C for 14 days. All subsequent experiments were initiated from these master cultures.

### 2.2 Host defence volatile chemical stock preparation

Eight host defence volatiles from lodgepole pine were used, including seven monoterpenes: (+)‐α‐pinene, (−)‐α‐pinene, (+)‐limonene, (−)‐limonene, 3‐carene, terpinolene and myrcene, and one phenylpropanoid, 4‐allylanisole. For each volatile, we prepared the concentration‐specific dosing stocks by dissolving the neat chemical in Polysorbate 80 (Tween 80; Thermo Fisher Scientific, Ottawa, ON, CAN). A set of stock solutions for each volatile was formulated to correspond to target exposure concentrations (ng mg^−1^) of 50, 100, 200, 400, 800, 1600, 3200 and 6400. Stock concentrations were back‐calculated (Supporting information Table S1) so that adding a fixed volume of stock to the culture medium would yield the intended target volatile concentration while maintaining a final Polysorbate 80 content of 0.5% (v/v). When these concentrations did not encompass the full inhibitory range (i.e. when all tested concentrations did not result in inhibition of <30% and/or >90%), additional concentrations were added at the lower and/or higher ends of the range to refine the dose–response curve. A solvent‐only stock (Polysorbate 80) also was prepared as a negative control. All stocks were vortex‐mixed until homogeneous, dispensed into amber glass vials with PTFE‐lined caps (ULINE, Milton, ON, Canada), filled to at least 3/4 of the vial volume to minimize headspace, and stored at −40 °C in the dark until use.

### 2.3 Fungal exposure and incubation

Each of the three fungi, *G. clavigera*, *L. longiclavatum* and *O. montium*, was exposed to a single volatile concentration described above. We prepared five independent replicates for each concentration in each fungal species. We dispensed 25 mL autoclaved potato dextrose broth (PDB; Becton, Dickinson and Co., Franklin Lakes, NJ, USA) into each 50‐mL borosilicate glass flask under aseptic conditions. The calculated volume of each monoterpene stock solution was added to achieve the designated concentration, and the mixture was gently inverted to ensure uniform mixing. We then transferred a 7‐mm‐diameter mycelial plug to each flask, cut from the actively growing edge of the master culture. We immediately sealed the flask with two layers of Parafilm sealing film (Amcor, Oshkosh, WI, USA) to minimize volatilization and contamination and incubated it in complete darkness at 25 °C for 96 h. Solvent controls containing 0.5% (v/v) Polysorbate 80 without any added compound were included for each fungal isolate.

### 2.4 Biomass collection and measurement

After 96 h of incubation, we filtered the culture medium to separate the fungal hyphae from the broth, carefully removed the mycelial plug, and gently rinsed the retained mycelium on the filter with ultrapure water to remove residual medium components. We then transferred the fungal mat onto a pre‐weighed quantitative filter paper (grade P4, 55 mm diameter; Fisherbrand, Pittsburgh, PA, USA) that had been dried in a freeze dryer for 48 h. Each loaded filter paper was rapidly immersed in liquid nitrogen for ≈30 s to terminate metabolic activity and preserve tissue structure. We then freeze‐dried the samples for 48 h and weighed them. Final fungal biomass was calculated as the difference between the dry weight (DW) of the filter–mycelium composite and the initial filter paper weight.

### 2.5 Dose–response modelling

We quantified fungal sensitivity to each compound by fitting classical dose–response models to individual replicate data. For each fungal species–compound combination, the percent inhibition for each replicate was calculated relative to the mean dry biomass of the corresponding solvent control (0.5% v/v Polysorbate 80) according to the equation:

$$I\left(\%\right)=100\times \left(1-\frac{B_{\text{treat}}}{{\bar{B}}_{\text{control}}}\right)$$

where $B_{\text{treat}}$ is the dry biomass (mg) of an individual replicate at a given concentration, and ${\bar{B}}_{\text{control}}$ is the mean biomass of the control group from the same experimental batch.

We fitted dose–response curves using the classical two‐parameter log‐logistic model, which provides a parsimonious and biologically interpretable description of monotonic dose–response relationships and is widely applied in toxicological analyses.^41^ This model assumes that the inhibitory response follows a sigmoidal trajectory, approaching complete inhibition at high concentrations and no inhibition at low concentrations. We fixed the lower and upper limits at 0 and 100, respectively, based on preliminary experiments showing that fungal growth was suppressed entirely at sufficiently high concentrations and indistinguishable from the solvent control at the lowest concentrations tested. We then estimated the slope (b) and the median inhibitory concentration ($IC_{50}$) as free parameters using the following functional form:

$$f\left(x\right)=\frac{100}{\left(1+\exp\left[b\left(\ln x-\ln IC_{50}\right)\right]\right)}$$

where x is the concentration of the tested compound (ng mg^−1^).

We visually inspected each fitted curve to ensure that the observed responses followed a monotonic trend consistent with the log‐logistic assumption. When the data showed nonmonotonic or irregular patterns (e.g. stimulation at low concentrations, plateaus at intermediate doses, or incomplete inhibition at the upper range), we considered the compound to deviate from the model assumption and excluded it from further curve‐fitting and IC estimation. We fitted all dose–response models in R (v4.5.1^42^) using the drc package.^41^ We evaluated model goodness‐of‐fit using pseudo‐*R* ^2^ values and derived standard errors (SE) from the parameter covariance matrix. When residual variance increased with concentration, we corrected for heteroscedasticity by applying inverse‐variance weighting. For each fitted model, we calculated the IC_50_ and IC_90_ (the concentrations causing 50% and 90% inhibition of fungal growth, respectively), along with their SEs and 95% confidence intervals (CIs). Smaller IC values indicate more substantial toxicity.

## 3 RESULTS

### 3.1 Dose–response model of Grosmannia clavigera

Initial tests using the eight‐point concentration (ng mg^−1^) series (50–6400) revealed that the inhibition range was not fully captured by the number of concentrations used. We therefore expanded the assays by adding additional concentrations to cover both low‐ and high‐end responses. Two lower concentrations (12.5 and 25) were added for 3‐carene, terpinolene, 4‐allylanisole and myrcene, whereas two higher concentrations (12 800 and 25 600) were added for (−)‐α‐pinene. No additional treatments were required for (±)‐limonene or (+)‐α‐pinene.

**Figure 1:**
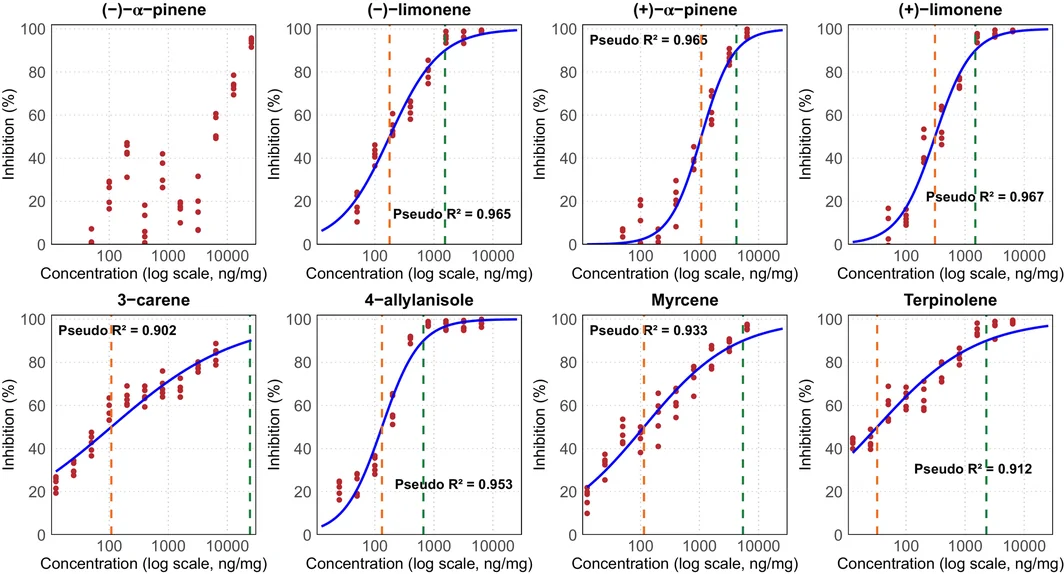
Dose–response relationships between growth inhibition of *G. clavigera* and the concentrations of host defence volatiles. Observed inhibition values (red points) and fitted log‐logistic curves (blue lines) are shown for each of the eight tested plant host defence volatiles. Fungal inhibition (%) was calculated relative to solvent controls (0.5% v/v Polysorbate 80). Vertical dashed lines indicate the estimated concentrations causing 50% (IC₅₀, orange) and 90% (IC₉₀, green) inhibition of fungal growth, respectively. The *x*‐axis is plotted on a logarithmic scale but labelled with actual concentration values (ng mg^−1^ of media). All panels share the same *x–y* scales for direct comparison of slope and toxicity among host defence volatiles. Pseudo‐*R* ^2^ values denote model fit quality.

Seven host defence volatiles, excluding (−)‐α‐pinene, produced monotonic inhibition patterns suitable for the constrained log–logistic model (lower and upper limits fixed at 0 and 100%, respectively; Fig. 1), and model fits were high (pseudo‐*R* ^2^ = 0.902–0.967). By contrast, (−)‐α‐pinene exhibited a nonmonotonic, irregular inhibition pattern, with suppression increasing up to 200 ng mg^−1^, followed by a decline and marked variability across mid‐range concentrations (200–3200). Beyond 3200, inhibition rose sharply, reaching near‐complete suppression only at the highest doses (≥25 600; Fig. 1).

Toxicity rankings based on IC₅₀ and IC₉₀ values for *G. clavigera* are presented in Fig. 1 and Table 1. IC₅₀: terpinolene (32.3) > 3‐carene (108.5) ≈ myrcene (114.8) > 4‐allylanisole (132.0) > (−)‐limonene (177.7) > (+)‐limonene (308.7) > > (+)‐α‐pinene (1071.2). IC₉₀: 4‐allylanisole (664.8 ng mg^−1^) > (+)‐limonene (1489.8) > (−)‐limonene (1544.1) > terpinolene (2289.7) > (+)‐α‐pinene (4219.1) > myrcene (5435.4) > > 3‐carene (24 226.8).

**Table 1:** Dose–response parameters of three fungal symbionts of the mountain pine beetle exposed to host defence volatiles

Compound	Fungal Species	Slope parameter (*b*)		IC₅₀				IC₉₀				
		Value	SE (slope)	Value	SE	95% CI lower	95% CI upper	Value	SE	95% CI lower	95% CI upper	Pseudo‐*R* ^2^
(−)‐α‐pinene	*Grosmannia clavigera*	N/A	N/A	N/A	N/A	N/A	N/A	N/A	N/A	N/A	N/A	N/A
	*Leptographium longiclavatum*	N/A	N/A	N/A	N/A	N/A	N/A	N/A	N/A	N/A	N/A	N/A
	*Ophiostoma montium*	−0.739	0.039	714.4	55.3	603.3	825.5	13 959.7	2372.9	9188.7	18 730.8	0.946
(−)‐limonene	*Grosmannia clavigera*	−1.016	0.050	177.7	9.1	159.3	196.2	1544.1	170.6	1198.8	1889.4	0.965
	*Leptographium longiclavatum*	−1.065	0.058	238.1	13.1	211.6	264.6	1873.2	231.4	1404.7	2341.6	0.962
	*Ophiostoma montium*	−1.388	0.128	470.9	34.9	400.3	541.6	2293.4	341.4	1602.2	2984.6	0.937
(+)‐α‐pinene	*Grosmannia clavigera*	−1.603	0.114	1071.2	53.6	962.7	1179.7	4219.1	454.5	3298.9	5139.2	0.965
	*Leptographium longiclavatum*	−1.407	0.133	1318.2	91.3	1133.5	1503.0	6285.2	963.7	4334.4	8236.1	0.934
	*Ophiostoma montium*	−1.292	0.097	826.1	52.3	720.2	932.0	4524.3	594.9	3320.1	5728.6	0.952
(+)‐limonene	*Grosmannia clavigera*	−1.396	0.084	308.7	15.8	276.6	340.8	1489.8	159.9	1166.0	1813.5	0.967
	*Leptographium longiclavatum*	−0.913	0.072	244.2	21.9	200.0	288.5	2707.3	508.9	1677.1	3737.5	0.909
	*Ophiostoma montium*	−0.923	0.069	349.6	30.0	288.9	410.3	3776.7	674.5	2411.2	5142.2	0.920
3‐carene	*Grosmannia clavigera*	−0.406	0.023	108.5	11.0	86.4	130.5	24 226.8	7266.5	9616.5	38 837.1	0.902
	*Leptographium longiclavatum*	−0.608	0.039	569.8	61.1	447.1	692.6	21 207.5	4979.3	11 195.9	31 219.1	0.904
	*Ophiostoma montium*	−1.299	0.070	382.5	18.5	345.1	419.9	2076.6	209.7	1652.0	2501.2	0.972
4‐allylanisole	*Grosmannia clavigera*	−1.359	0.103	132.0	7.7	116.4	147.6	664.8	81.3	500.9	828.7	0.953
	*Leptographium longiclavatum*	−1.265	0.094	126.9	7.5	111.8	142.0	721.1	99.2	520.4	921.9	0.941
	*Ophiostoma montium*	−1.733	0.152	207.2	11.3	184.3	230.0	736.1	82.0	570.1	902.0	0.953
Myrcene	*Grosmannia clavigera*	−0.570	0.029	114.8	9.8	95.2	134.4	5435.4	1095.7	3232.3	7638.6	0.933
	*Leptographium longiclavatum*	−1.054	0.072	187.2	11.5	163.8	210.5	1504.7	224.0	1051.3	1958.1	0.945
	*Ophiostoma montium*	−0.918	0.063	598.6	46.8	503.7	693.4	6552.0	1152.2	4219.4	8884.5	0.932
Terpinolene	*Grosmannia clavigera*	−0.516	0.030	32.3	3.5	25.3	39.3	2289.7	477.7	1329.3	3250.1	0.912
	*Leptographium longiclavatum*	−0.795	0.050	210.0	16.5	176.6	243.3	3324.9	578.2	2154.3	4495.5	0.929
	*Ophiostoma montium*	−1.424	0.143	425.2	30.2	363.8	486.6	1989.1	338.2	1301.8	2676.4	0.940

Parameter estimates from two‐parameter log‐logistic models describing the inhibition of fungal growth as a function of compound concentration. Slope parameter represents the steepness of the dose–response curve. IC₅₀ and IC₉₀, estimated concentrations (ng mg^−1^ of medium) that caused 50% and 90% inhibition of dry biomass, respectively. SE and 95% CI were derived using the delta method based on the parameter covariance matrix on the log‐transformed scale.^41^ Pseudo‐*R* ^2^ values indicate model goodness‐of‐fit. “N/A" denotes cases where a model could not be fitted owing to nonmonotonic or stimulatory responses.

Differences between IC₅₀ and IC₉₀ were associated primarily with variation in slope parameters (*b*; Table 1). Host defence volatiles with shallow slopes, such as 3‐carene (|*b*| = 0.406) and myrcene (|*b*| = 0.570), showed large gaps between IC₅₀ and IC₉₀ values, reflecting gradual inhibition across a broad concentration range. By contrast, host defence volatiles with steep slopes (|*b*| > 1), including (+)‐α‐pinene, (+)‐limonene, 4‐allylanisole and (−)‐limonene, reached near‐complete inhibition rapidly once concentrations exceeded the threshold range. Noticeable differences were observed between enantiomers of α‐pinene and limonene, exhibiting distinct slope parameters and inhibitory concentrations.

### 3.2 Dose–response model of *Leptographium longiclavatum*

Initial tests using the eight‐point concentration series revealed that the inhibition range was not fully captured for several host defence volatiles. We therefore expanded the assays for *L. longiclavatum* by adding additional concentration groups to cover high‐end responses for some host defence volatiles. Two higher concentrations (12 800 and 25 600) were added for 3‐carene and (−)‐α‐pinene. No extensions were required for the remaining host defence volatiles.

**Figure 2:**
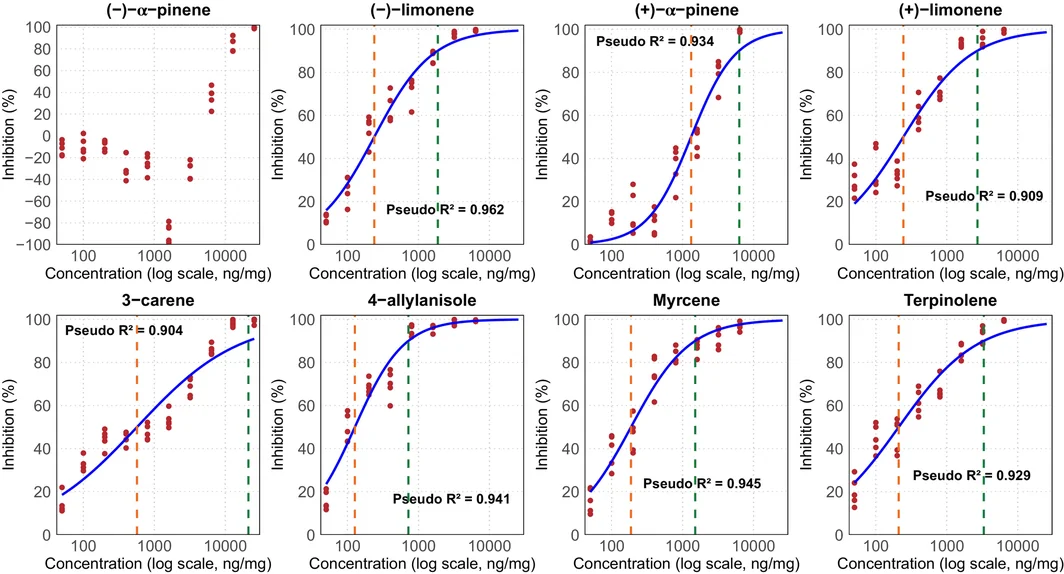
Dose–response relationships between growth inhibition of *L. longiclavatum* and the concentrations of host defence volatiles. Observed inhibition values (red points) and fitted log‐logistic curves (blue lines) are shown for each of the eight tested host defence volatiles. Fungal inhibition (%) was calculated relative to solvent controls (0.5% v/v Polysorbate 80). Vertical dashed lines indicate the estimated concentrations causing 50% (IC₅₀, orange) and 90% (IC₉₀, green) inhibition of fungal growth, respectively. The *x*‐axis is plotted on a logarithmic scale but labelled with actual concentration values (ng mg^−1^ of media). All panels share the same *x–y* scales for direct comparison of slope and toxicity among host defence volatiles, except for (−)‐α‐pinene, which exhibited negative inhibition values (growth stimulation) and was therefore plotted with an expanded y‐axis range. Pseudo‐*R* ^2^ values denote model fit quality.

Similar to *G. clavigera*, the same seven host defence volatiles produced monotonic inhibition patterns suitable for the constrained log–logistic model (lower and upper limits fixed at 0 and 100%, respectively; Fig. 2) in *L. longiclavatum*, with high model fits (pseudo‐*R* ^2^ = 0.904–0.962). By contrast, (−)‐α‐pinene exhibited a nonmonotonic pattern characterized by negative inhibition (apparent growth stimulation) that progressively intensified with increasing concentration up to 1600, then diminished toward neutrality, and ultimately shifted to a rapid increase in inhibition beyond 3200, reaching near‐complete suppression at ≥25 600 ng mg^−1^ (Fig. 2). Consequently, (−)‐α‐pinene was not modelled here.

Toxicity rankings based on IC₅₀ and IC₉₀ values for *L. longiclavatum* are presented in Fig. 2 and Table 1. IC₅₀: 4‐allylanisole (126.9) > myrcene (187.2) > terpinolene (210.0) > (−)‐limonene (238.1) ≈ (+)‐limonene (244.2) > 3‐carene (569.8) > > (+)‐α‐pinene (1318.2). IC₉₀: 4‐allylanisole (721.1 ng mg^−1^) > myrcene (1504.7) > (−)‐limonene (1873.2) > (+)‐limonene (2707.3) > terpinolene (3324.9) > (+)‐α‐pinene (6285.2) > > 3‐carene (21 207.5).

Differences between IC₅₀ and IC₉₀ were moderate but compound‐specific, primarily reflecting slope variation (*b*; Table 1). Myrcene and 4‐allylanisole consistently showed the highest toxicity across both metrics, whereas 3‐carene, with the shallowest slope (|*b*| = 0.608), exhibited a pronounced shift from moderate to the least toxic position owing to its slow, gradual inhibition response. Terpinolene and the two limonene enantiomers also showed minor rank reversals, consistent with differences in slope steepness. Noticeable enantiomeric differences were observed in *L. longiclavatum*: (+)‐ and (−)‐limonene showed comparable IC₅₀ values but diverged in IC₉₀ and slope magnitudes, whereas (+)‐α‐pinene was substantially less toxic than its (−)‐form, consistent with the latter's irregular, nonmonotonic response pattern.

### 3.3 Dose–response model of *Ophiostoma montium*

**Figure 3:**
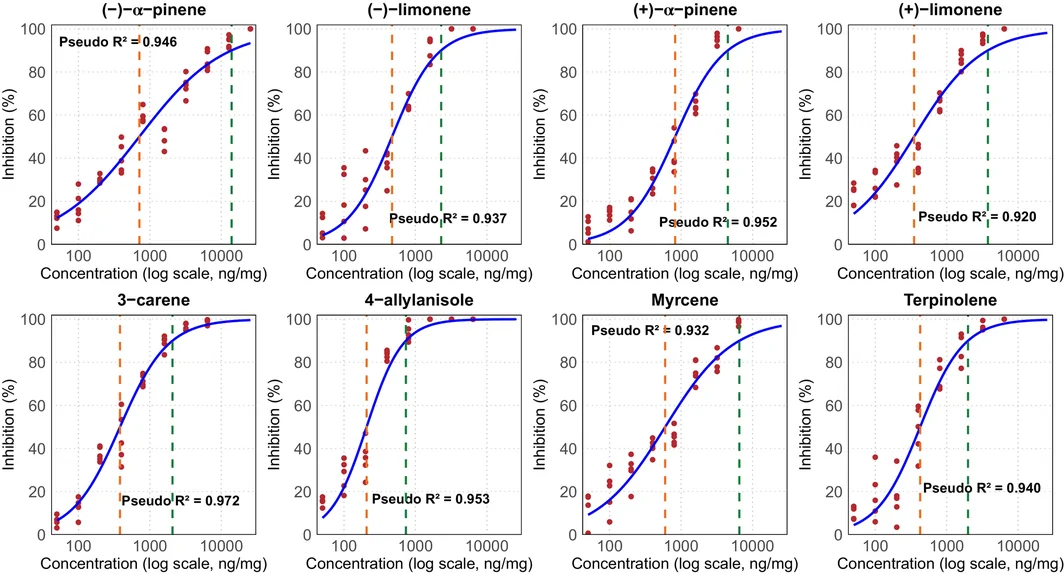
Dose–response relationships between growth inhibition of *O. montium* and the concentrations of host defence volatiles. Observed inhibition values (red points) and fitted log‐logistic curves (blue lines) are shown for each of the eight tested host defence volatiles. Fungal inhibition (%) was calculated relative to solvent controls (0.5% v/v Polysorbate 80). Vertical dashed lines indicate the estimated concentrations causing 50% (IC₅₀, orange) and 90% (IC₉₀, green) inhibition of fungal growth, respectively. The *x*‐axis is plotted on a logarithmic scale but labelled with actual concentration values (ng mg^−1^ of media). All panels share the same *x–y* scales for direct comparison of slope and toxicity among host defence volatiles. Pseudo‐*R* ^2^ values denote model fit quality.

Initial tests using the eight‐point concentration series sufficiently captured the inhibition range for all host defence volatiles in *O. montium*. Therefore, no additional concentration groups were required. Unlike the other two fungal symbionts, all eight host defence volatiles produced monotonic inhibition suitable for the constrained log–logistic model (limits fixed at 0/100%; Fig. 3), with high fits (pseudo‐*R* ^2^ = 0.920–0.972).

Toxicity rankings based on IC₅₀ and IC₉₀ values for *O. montium* were presented in Fig. 3 and Table 1. IC₅₀: 4‐allylanisole (207.2) > (+)‐limonene (349.6) > 3‐carene (382.5) > terpinolene (425.2) > (−)‐limonene (470.9) > myrcene (598.6) > (−)‐α‐pinene (714.4) > (+)‐α‐pinene (826.1). IC₉₀: 4‐allylanisole (736.1 ng mg^−1^) > terpinolene (1989.1) > 3‐carene (2076.6) > (−)‐limonene (2293.4) > (+)‐limonene (3776.7) > (+)‐α‐pinene (4524.3) > myrcene (6552.0) > (−)‐α‐pinene (13 959.7).

Differences between IC₅₀ and IC₉₀ mainly reflected variation in slope steepness (*b*; Table 1). Host defence volatiles with steep slopes, such as 4‐allylanisole (|*b*| = 1.733), terpinolene (1.424) and 3‐carene (1.299), maintained strong inhibition across concentration ranges, resulting in small IC₅₀–IC₉₀ gaps and stable toxicity rankings. By contrast, host defence volatiles with shallower slopes, particularly myrcene (|*b*| = 0.918), (+)‐limonene (0.923) and (−)‐α‐pinene (0.739), required substantially higher concentrations to achieve near‐complete inhibition, resulting in lower relative toxicity at the IC₉₀ threshold. Furthermore, enantiomeric contrasts were evident. For limonene, (+) had a lower IC₅₀ than (−) but, owing to its shallower slope, a higher IC₉₀ (3776.7 *versus* 2293.4). For α‐pinene, (−) appeared more toxic at IC₅₀ (714.4 *versus* 826.1) but was far less toxic at IC₉₀ (13 959.7 *versus* 4524.3), which is the result of the much shallower slope of (−)‐α‐pinene.

## 4 DISCUSSION

We tested the toxicity of eight host defence volatiles on fungal biomass for three symbiotic fungi of MPB. The degree of inhibition varied across volatile compounds and fungal species. *Grosmannia clavigera* exhibited the strongest overall growth inhibition, whereas *O. montium* was the most tolerant. Except for (−)‐α‐pinene for *L. longiclavatum*, all volatiles inhibited biomass production of all three fungi. Among volatiles tested, 4‐allylanisole caused strong inhibition, whereas (−)‐α‐pinene was overall less toxic. Enantiomeric forms differed in inhibition level. Several compound–fungus pairs showed large differences between IC₅₀ and IC₉₀, ranging from gradual inhibition to near‐complete inhibition within a narrow concentration range. Notably, (−)‐α‐pinene showed irregular, nonmonotonic inhibition in both *G. clavigera* and *L. longiclavatum*, with variable inhibition in the former and clear low‐dose stimulation followed by inhibition in the latter. Overall, we found distinct toxicity profiles across host defence volatiles and fungi, with characteristic patterns of sensitivity, slope and variability that describe how each fungal species responds to increasing concentrations of volatile compounds and may potentially influence their persistence and functional roles during host colonization of MPB–fungal symbionts.

### 4.1 Fungal species exhibit varying toxicity responses to host defence volatiles

The variation in inhibition among the three fungal symbionts indicates that tolerance to host defence volatiles is not uniform across species and that reflects species‐specific differences in physiological and biochemical mechanisms that mitigate the toxicity. Among the three species, *G. clavigera* is considered the most aggressive, rapidly colonizing host‐tree phloem. Its genome encodes diverse genes involved in detoxification and oxidative stress tolerance, including cytochrome P450 monooxygenases, ABC transporters and antioxidant enzymes.^10^, ^32^ Both phenotypic and molecular evidence indicate that *G. clavigera* can utilize host defensive monoterpenes as carbon sources for growth.^32^, ^33^, ^34^ By contrast, *O. montium* tends to persist in conditions where host defences are weaker or have subsided after colonization of host tissues with more aggressive fungal species such as *G. clavigera*.^12^ It grows more slowly and is often replaced by more aggressive symbionts when host defences are strong.^2^, ^8^, ^16^ *Leptographium longiclavatum* shows intermediate characteristics between *G. clavigera* and *O. montium*.^14^ We propose that fungal symbionts occupy distinct ecological niches within the beetle system, where their tolerance of defence volatiles may be able to explain their relative roles during different stages of host colonization.^35^, ^43^

### 4.2 Fungal tolerance to host volatiles influences host‐specific adaptation

Within a given defence volatile, the difference between its IC₅₀ and IC₉₀ indicates how rapidly growth inhibition increases as concentration increases. Therefore, two defence volatiles with similar IC₅₀ values can still differ substantially in their IC₉₀ values, and in how close they approach complete growth inhibition at high exposure. Such variation has a strong ecological relevance. When bark beetles initiate a host colonization, hosts rely on constitutive defences to stop the attack. However, as the attack continues, induced defences become the primary mechanism defending trees. The main difference between constitutive and induced defences is that the latter is more toxic and thus lethal to the bark beetle–fungi symbioses.^9^, ^18^, ^19^, ^44^, ^45^, ^46^ We suspect that those fungi can grow early under low volatile exposure, but even with similar IC₅₀ values, volatiles that reach near‐complete inhibition quickly at elevated concentrations can shut down hyphal growth as resin production increases, whereas volatiles with more gradually increasing toxicity may be better tolerated and allow fungal persistence at high concentrations.

Furthermore, pine species differ markedly in their host defence volatile constituents; hence, the chemical environments MPB–fungal symbionts experience differ during host colonization. Ponderosa pine, one of MPB's ancestral hosts, is dominated by 3‐carene.^47^, ^48^ By contrast, jack pine, a novel host in MPB's expanded range, contains higher proportions of α‐pinene, particularly (+)‐α‐pinene.^29^ Such compositional differences among different host tree species may differentially affect MPB and fungal symbionts. We show that 3‐carene weakly inhibited all three fungi, producing slow, incomplete inhibition with shallow IC₅₀ and IC₉₀ dose–response slopes. This may suggest that, despite elevated chemical stress, fungi remain relatively tolerant to some host defence volatiles typical of their ancestral hosts, reflecting long‐term co‐adaptation within historical host conditions. Interestingly, (+)‐α‐pinene showed only moderate overall toxicity but a steep inhibition slope, suggesting that although fungi are not highly sensitive to this monoterpene at low concentrations, rapid suppression occurs once the tolerance threshold level is reached. Considering that the shift in monoterpene composition has been linked to the successful host range expansion of MPB,^27^ our research shows that (+)‐α‐pinene can contribute to jack pine chemical resistance against MPB‐associated fungi, although α‐pinene also is widely discussed as a pheromone precursor that may facilitate MPB aggregation in jack pine.^20^, ^24^

Notably, the enantiomer‐specific inhibition patterns we observed suggest adaptive biochemical specialization. This indicates that stereochemistry affects fungal metabolism, a process likely to be via enantioselective detoxification or membrane interactions.^49^, ^50^, ^51^ Such tolerance may reflect long‐term adaptation to host‐specific enantiomeric profiles that have been reported to vary among pine species and regions,^26^, ^27^, ^28^, ^52^ influencing fungal performance and beetle fitness in distinct chemical environments.

### 4.3 Fungal tolerance to host defence volatiles reshapes the host–beetle chemical interactions

Host defence volatiles serve multiple ecological functions; the most fundamental of which is toxicity. Their inhibitory effects on MPB have been well‐documented.^21^, ^22^, ^29^ Notably, the host defence volatiles most toxic to MPB were not always the strongest inhibitors of its symbiotic fungi, and *vice versa*.^35^ The diverse array of host defence chemicals appears to have evolved to act on the beetle–fungus complex through distinct and partly nonoverlapping physiological targets, consistent with the ‘interaction diversity’ hypothesis.^40^ Defence volatiles that strongly impair beetle performance are likely to act through neurotoxic or respiratory disruption pathways^22^, ^53^, ^54^ and are comparatively less effective against fungal symbionts, which possess alternative membrane compositions and detoxification mechanisms.^10^, ^32^ Conversely, compounds that suppress fungal growth, by disrupting membrane integrity, enzyme function, or primary metabolism,^55^, ^56^, ^57^ tend to exert weaker effects on insects, revealing mechanistic trade‐offs in host chemical defences across symbiotic partners.^35^ In our study, strongly antifungal host defence volatiles such as terpinolene and 4‐allylanisole complement those that primarily deter or intoxicate beetles, such as limonene and 3‐carene,^21^, ^22^ together forming a multitarget, chemically integrated defence system that is difficult for either partner to fully circumvent. This functional complementarity among host defence volatiles may help explain why pine oleoresin contains many host defence volatiles at moderate concentrations rather than relying on a single dominant toxin: chemical diversity itself serves as a defence strategy, allowing the tree to constrain attackers with overlapping yet distinct modes of action.^58^, ^59^, ^60^

The toxicity profiles of these host defence volatiles suggest that host defence volatiles also participate in other ecological interactions beyond direct defence. In both *G. clavigera* and *L. longiclavatum*, (−)‐α‐pinene showed nonmonotonic, concentration‐dependent responses, whereas *O. montium* showed a more typical inhibitory pattern. Such irregular, biphasic reactions imply that (−)‐α‐pinene may act as a metabolizable substrate at sublethal levels but as a toxicant when accumulated. Given that MPB depends on (−)‐α‐pinene as a pheromone precursor,^36^, ^61^ both the beetle and its symbionts must maintain partial tolerance to this compound to maintain effective communication. The nonmonotonic fungal responses that we observed may thus represent a biochemical accommodation to a compound that simultaneously mediates host defence and could influence beetle signalling. Interestingly, 4‐allylanisole, which is a reported pheromone inhibitor,^29^ was the most toxic to all three fungi. This suggests that 4‐allylanisole may act as a broad‐spectrum defence by interfering with pheromone‐mediated attack coordination and limiting symbiont establishment.

### 4.4 Shortcomings and future direction

Our study tested species‐specific toxicity profiles of MPB‐associated fungi under single‐compound exposure. In nature, however, fungi encounter complex mixtures of such defence volatiles and phenolics that interact synergistically or antagonistically to shape tree defence capacity.^62^ Additionally, symbiotic fungi release their own volatile organic compounds that can alter host and beetle chemical environments, mediating both defence and beetle communication.^63^ Future work should examine multicomponent interactions and fungal volatiles cross‐effects to better understand how fungal metabolism, host defence, and beetle signalling coevolve within this chemically integrated system.

## FUNDING INFORMATION

This research is funded by the Natural Sciences and Engineering Research Council of Canada‐Discovery to NE and cofounded by China Scholarship Council Undergraduate Research Internship Program to DZ (no. 202406170318) and XZ (no. 202406170321).

## CONFLICT OF INTEREST

The authors declare that they have no known competing financial interests or personal relationships that could have appeared to influence the work reported in this paper.

## AUTHOR CONTRIBUTIONS

Duo Zhang: Methodology, Validation, Formal analysis, Investigation, Writing – review & editing, Project administration. Yanzhuo Liu: Conceptualization, Methodology, Validation, Formal analysis, Investigation, Writing – original draft, Writing – review & editing, Visualization, Supervision, Project administration. Xinyi Zhao: Methodology, Investigation. Guncha Ishangulyyeva: Methodology, Investigation, Project administration, Writing – review & editing. Nadir Erbilgin: Conceptualization, Methodology, Writing – original draft, Writing – review & editing, Supervision, Project administration, Funding acquisition.

## Supporting information


**Table S1.** Dosing calculations for the preparation of compound‐specific stock and working solutions. The top panel lists the standard density (g mL^−1^) and standard purity (fraction of active ingredient) of each host defence volatile standard. The middle block (‘To prepare the stock solutions…’) provides, for each compound and target exposure concentration, the volume (μL) of the chemical standard added to 2.5 mL Polysorbate 80 to prepare a dosing stock solution (values calculated to three decimal places). The bottom block (‘In the actual experiments…’) lists the volume (μL) of each prepared stock solution added to 25 mL PDB medium to achieve the target final concentration (values calculated to three decimal places). Target concentrations are expressed as ng mg^−1^ of medium.

## Data Availability

The data underlying this article will be shared on reasonable request to the corresponding author.
