# Supplementary material for: Dose–response modelling uncovers compound‐specific inhibitory effects of host defence volatiles on mountain pine beetle fungal symbionts
Source: Pest Manag Sci. 2026 Jun 25;82(10):10241–50. doi: 10.1002/ps.71060 (PMC13569354; doi:10.1002/ps.71060)
Supplement: Supplementary file 1 [file PS-82-10241-s001.docx]

**Supplementary Information**

**Dose–response modelling uncovers compound-specific inhibitory effects of host defence volatiles on mountain pine beetle fungal symbionts**

Duo Zhang^1,2,#^, Yanzhuo Liu^1,#,*^, Xinyi Zhao^1,2^, Guncha Ishangulyyeva^1^, and Nadir Erbilgin^1^

^1^ Department of Renewable Resources, University of Alberta, Edmonton, Alberta, Canada.

^2^ College of Veterinary Medicine, Jilin University, Changchun, Jilin, China

^#^ Duo Zhang and Yanzhuo Liu contributed equally to this work.

^*^ Correspondence concerning this article should be addressed to Yanzhuo Liu, Department of Renewable Resources, 4-42 Earth Science Building, 115 Street & Saskatchewan Drive, University of Alberta, Edmonton, Alberta T6G 2E3, Canada. Email: [yanzhuo@ualberta.ca](mailto:yanzhuo@ualberta.ca)

**Table S1.** Dosing calculations for the preparation of compound-specific stock and working solutions. The top panel lists the standard density (g mL⁻¹) and standard purity (fraction of active ingredient) of each host defence volatile standard. The middle block (“To prepare the stock solutions…”) provides, for each compound and target exposure concentration, the volume (µL) of the chemical standard added to 2.5 mL of Polysorbate 80 to prepare a dosing stock solution (values calculated to three decimal places). The bottom block (“In the actual experiments…”) lists the volume (µL) of each prepared stock solution added to 25 mL of PDB medium to achieve the target final concentration (values calculated to three decimal places). Target concentrations are expressed as ng mg⁻¹ of medium.

|  | | (-)-α-pinene | (+)-α-pinene | (-)-limonene | (+)-limonene | 3-carene | Terpinolene | 4-allylanisole | Myrcene |
| --- | --- | --- | --- | --- | --- | --- | --- | --- | --- |
| standard density (g/mL) | | 0.858 | 0.858 | 0.8411 | 0.8411 | 0.864 | 0.861 | 0.965 | 0.794 |
| standard purity | | 0.81 | 0.8 | 0.96 | 0.97 | 0.9 | 0.85 | 0.98 | 0.9 |
|  | | | | | | | | | |
| To prepare the stock solutions, the following volumes (µL) of standard compounds were added to 2.5 mL of Polysorbate 80, respectively. | | | | | | | | | |
| Target final concentration in media (ng/mg) | 12.5 | 9.21 | 9.32 | 7.93 | 7.84 | 8.23 | 8.74 | 6.77 | 8.96 |
|  | 25 | 18.42 | 18.65 | 15.85 | 15.69 | 16.46 | 17.49 | 13.53 | 17.91 |
|  | 50 | 36.84 | 37.30 | 31.70 | 31.38 | 32.92 | 34.98 | 27.07 | 35.82 |
|  | 100 | 73.67 | 74.59 | 63.41 | 62.76 | 65.84 | 69.96 | 54.14 | 71.65 |
|  | 200 | 147.34 | 149.18 | 126.82 | 125.51 | 131.69 | 139.92 | 108.28 | 143.30 |
|  | 400 | 294.68 | 298.37 | 253.64 | 251.02 | 263.37 | 279.84 | 216.56 | 286.59 |
|  | 800 | 589.37 | 596.74 | 507.27 | 502.04 | 526.75 | 559.68 | 433.12 | 573.19 |
|  | 1600 | 1178.74 | 1193.47 | 1014.54 | 1004.09 | 1053.50 | 1119.36 | 866.24 | 1146.38 |
|  | 3200 | 2357.48 | 2386.95 | 2029.09 | 2008.17 | 2107.00 | 2238.71 | 1732.47 | 2292.75 |
|  | 6400 | 4714.96 | 4773.89 | 4058.18 | 4016.34 | 4213.99 | 4477.42 | 3464.95 | 4585.50 |
|  | 12800 | 9429.91 | 9547.79 | 8116.36 | 8032.68 | 8427.98 | 8954.84 | 6929.89 | 9171.00 |
|  | 25600 | 18859.82 | 19095.57 | 16232.71 | 16065.36 | 16855.97 | 17909.68 | 13859.79 | 18342.01 |
|  | | | | | | | | | |
| In the actual experiments, the following volumes (µL) of the prepared stock solutions were added to 25 mL of PDB medium. | | | | | | | | | |
| Target final concentration in media (ng/mg) | 12.5 | 125.46 | 125.47 | 125.40 | 125.39 | 125.41 | 125.44 | 125.34 | 125.45 |
|  | 25 | 125.92 | 125.93 | 125.79 | 125.78 | 125.82 | 125.87 | 125.68 | 125.90 |
|  | 50 | 126.84 | 126.86 | 126.59 | 126.57 | 126.65 | 126.75 | 126.35 | 126.79 |
|  | 100 | 128.68 | 128.73 | 128.17 | 128.14 | 128.29 | 128.50 | 127.71 | 128.58 |
|  | 200 | 132.37 | 132.46 | 131.34 | 131.28 | 131.58 | 132.00 | 130.41 | 132.16 |
|  | 400 | 139.73 | 139.92 | 137.68 | 137.55 | 138.17 | 138.99 | 135.83 | 139.33 |
|  | 800 | 154.47 | 154.84 | 150.36 | 150.10 | 151.34 | 152.98 | 146.66 | 153.66 |
|  | 1600 | 183.94 | 184.67 | 175.73 | 175.20 | 177.67 | 180.97 | 168.31 | 182.32 |
|  | 3200 | 242.87 | 244.35 | 226.45 | 225.41 | 230.35 | 236.94 | 211.62 | 239.64 |
|  | 6400 | 360.75 | 363.69 | 327.91 | 325.82 | 335.70 | 348.87 | 298.25 | 354.28 |
|  | 12800 | 596.50 | 602.39 | 530.82 | 526.63 | 546.40 | 572.74 | 471.49 | 583.55 |
|  | 25600 | 1067.99 | 1079.78 | 936.64 | 928.27 | 967.80 | 1020.48 | 817.99 | 1042.10 |

***Calculations and assumptions:*** Delivered mass was calculated as density × purity × volume. Volumes were back-calculated to ensure that (1) the final Polysorbate 80 content in all treatments and solvent controls was 0.5% (v/v) at the exposure step, and (2) the specified target series (12.5–25600 ng mg⁻¹) was obtained while maintaining an identical solvent background. Densities correspond to supplier-reported values at room temperature (≈ 20–25 °C). Standard purity refers to the reagent's purity as provided by the supplier.
